# Quality Evaluation of New Types of Layered Composites for Flooring Materials

**DOI:** 10.3390/ma17081892

**Published:** 2024-04-19

**Authors:** Sylwia Olenska, Piotr Beer

**Affiliations:** Institute of Wood Sciences and Furniture, Warsaw University of Life Sciences-SGGW, Nowoursynowska 159, 02-776 Warsaw, Poland; piotr_beer@sggw.edu.pl

**Keywords:** Shewhart control charts, quality, composite, flooring, veneer, peeling

## Abstract

The need, or even the obligation, to take care of the natural environment compels a search for new technological solutions, or for known solutions to be adapted to new applications. The maxim is ‘don’t harm, but improve the world for future generations’. In the wood industry in particular, given that it is based on a natural raw material, we must look for ecological solutions. Trees grow, but the demand for wood exceeds the volume of tree growth. In industrial manufacturing, one of the ways to make full use of wood is through chipless processing, which occurs during rotary cutting (peeling). In addition, wood is a natural material, each fragment of which has a range of properties. In addition, wood defects in quality manipulation generate a lot of waste. The aim of this study was to analyse the quality effect of the tested layered composites for flooring materials on production application. The practical purpose was to exchange actual sawing-based production for chipless production. The composite base layers were made of pine wood (*Pinus* L.) veneers with differing quality classes. The samples were subjected to three-point bending tests to calculate the moduli of elasticity and stiffness, which are the most important parameters. Because both analysed parameters describe product quality, the analyses were based on the creation of Shewhart control charts for each parameter. In theory, these control charts are tools for analysing whether the production process is stable and yields predictable results. To have full control over the process, five elements have to be applied: central line (target), two types of control lines (upper and lower) and two types of specification lines (upper and lower). New types of layered composites for flooring may be applied to production once verified using Shewhart control charts. It turns out that it is possible to produce the base layer of the flooring materials using the rotary cutting (peeling) method without having to analyse the quality of the raw material. This is a way to significantly increase the efficiency of production in every element of manufacturing.

## 1. Introduction

One of the very first elements of civil engineering, common for people throughout recorded history, is flooring. A floor is defined as a building part aimed at finishing off horizontal partitions [1]. From prehistoric times to the present day, man has always needed materials to walk on. The only differences are the technological possibilities that producers are able to offer their customers. The first known floors were the usual flattened out threshing floors. The main assumption of this was to protect bare feet against low temperatures. As time went by, people started to look at the aesthetic parameters of flooring and the comfort of use. As a result, dirt floors became stone flooring, and after that, they were manufactured using various species of wood [2]. Initially, the wood used to produce floors was selected for its visual properties. However, with further developments in human awareness, and therefore, technology, attention began to be paid to the physical and mechanical properties of floors in order to improve their durability, and consequently, their quality.

Nowadays, the main flooring materials made of solid wood are produced using cutting processes with circular saws or frame saws. Then, composites are produced from the obtained material by gluing them in layers [3,4,5]. Composites tend to be composed of two or three separate layers [6,7,8]. Plywood for floors is mainly used as an underlay material. In the case of the base layer of floor panels, changing the machining method from sawing to rotary cutting (peeling) would bring great benefits, especially in efficiency, as it is chipless cutting. This is the most effective process of using raw materials to produce the intended product [9]. Having wide, wooden sheets also allows them to be folded at a cross angle. This type of no-waste production is fully in line with sustainable industry, the action plans for a circular economy and the new European Bauhaus [10,11,12], and in particular, with main objective point 22, which:

“Stresses the importance of transforming, upgrading and retrofitting the existing building stock, including poorly planned and constructed buildings erected by totalitarian regimes, of applying nature-based solutions such as wood and of reducing waste and increasing durability, re-usability and circularity in the built environment; insists that this should include favouring renovation and adaptive re-use over demolition and new builds, as appropriate, removing barriers related to the handling and transport of waste as well as raising people’s awareness about embodied and stored carbon in materials to enable them to make informed choices”.

However, questions have arisen regarding the quality of the veneers obtained this way. In the sawing process, the quality selection of each element can be achieved quite easily. In the rotary cutting process, however, the quality selection is more difficult and would be ineffective, in the case of flooring materials.

The question arises whether veneer quality control in rotary cutting is really necessary. Production without the need for quality control and the certainty of receiving a high-quality finished product is invaluable. Such production can be achieved through layered composites for flooring materials with veneers [13]. However, it is necessary to check how veneers of different quality affect the mechanical properties of the composites for the flooring materials obtained from them. If veneers are used as load-bearing layers, any knots, especially loose knots, are substantial defects. Their diameter can range from a few millimetres to several centimetres. The standards PN-92/D-95017 [14] and PN-92/D-95008 [15] divide logs that are basic materials for natural veneer producers into four classes, A, B, C and D, depending on the share and size of the knots. It should be noted that Polish Standards for wood raw materials have been used on a voluntary basis since 1999, as is the case in the European Union [16]. However, the technical conditions that the sold wood must meet are specified by the General Director of National Forests [17]. The negative influence of knots is manifested in the lowering of the tensile, bending and compression strength along the fibres and the modulus of elasticity [18]. Mechanical tests of flooring materials [19] are not performed as often as exposures to temperature and humidity [20], hardness tests [13,21,22], top-layer examinations after material modifications [23,24,25] or tests regarding the in situ polymerisation of active monomers [26]. The research described in this paper examined the impacts of the structure of and the defects in wood on the surface quality of wood veneers. The results revealed that the presence of defects does not affect the roughness of the veneers and does not increase either the processing requirements of veneer sheets before finishing or the respective production cost of veneers and veneer-based wood panels [27]. The influence of defects in wood, and the veneers obtained from it, on the quality of the finished products was also examined. The tests performed can be used to evaluate the basic properties of veneers and plywood as functions of log temperature, which will lead to a better understanding of the properties of final panel products [28]. This will also shed light on the veneer compression process, which can be considered as an alternative method to improving both the physical and mechanical properties of experimental plywood panels used for building applications. [29]. Mechanical tests of composites for flooring materials, such as the modulus of elasticity in elastic deformation and also in dynamic and fatigue tests, as well as stiffness and static bending strength, indicate the possibility of mixing wood quality classes and still meeting technical requirements. This indicates the direction and manner of using all the wood as it is. Defects in growing wood are difficult to predict, and it is impossible to predict where they will occur. Despite this inconvenience, wood should be used in the most rational manner possible [30]. However, combining mechanical tests with analyses of the production possibilities of flooring materials is a new subject. The Shewhart control chart is a statistical method that uses data from empirical studies. While this method has rarely been applied in scientific research concerning wood technology, quality control is very often used in the industry. Therefore, implementing the Shewhart control chart concept and incorporating it into research on flooring materials is a valuable direction that may lead to future production progress and a comprehensive and flexible system for achieving, maintaining and maximising success [31].

The aim of this study was to analyse the quality effects of the tested layered composites for flooring materials on the production application. The utilitarian purpose was to exchange actual sawing-based production for chipless production that does not need qualitative manipulation. Therefore, this research was intended to determine the specific and direct application of the rotary cutting (peeling) method, which increases the yield and effectiveness of wood use.

## 2. Materials and Methods

### 2.1. Materials

**Figure 1:**
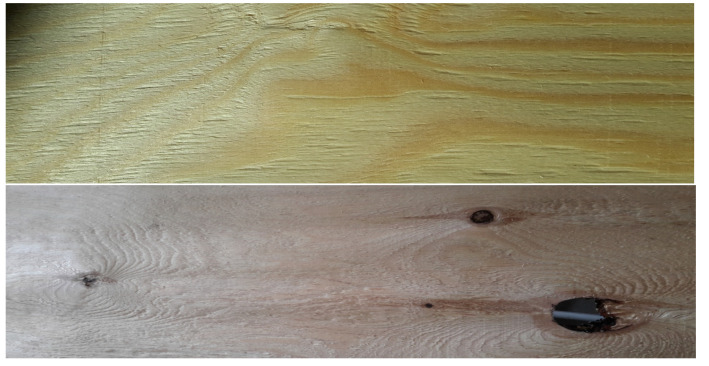
Examples of veneers in classes A (**top**) and D (**bottom**).

The materials to be tested were layered composites for flooring panels. The base layers of the flooring panels were made of three Scots pine (*Pinus sylvestris* L.) quality classes in accordance with standard EN 1927-2:2008 [32]. Pine logs with a density of 700–800 kg/m^3^ were rotary cut in order to obtain veneers. Those veneers were then divided into A, B/C and D classes, and 10 samples from each quality class were used for testing. Figure 1 presents examples of veneer classes A and D, which were used to produce the base layer of flooring materials.

**Figure 2:**
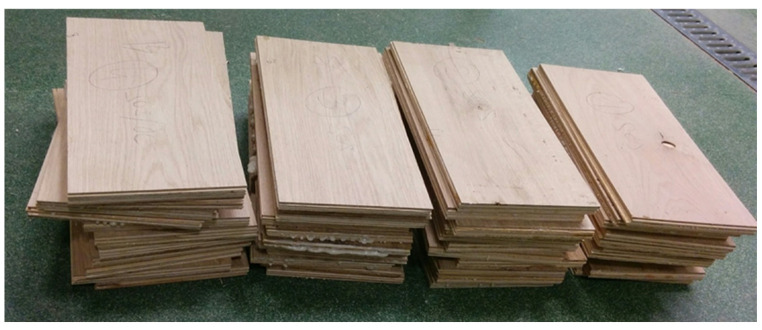
Samples prepared for static bending tests.

For the purposes of the qualitative analysis, classes B and C were combined. This is because it is difficult to separate them in real production conditions. Individual layers of veneers were placed in the inner layer at an angle of 90 degrees to each other (as in plywood). A urea–formaldehyde adhesive was used to bond the composite elements. The pressing parameters were set to the following specifications: the adhesive application was 200 g/m^2^, the temperature was 120 °C, the time was 60 s (the adhesive contained an additional 10% of the hardener) and the pressure was 1.2 MPa. The type of adhesive and the process parameters were recommended by a plywood manufacturer and are used in production. Once glued, the composites were air conditioned in a room at a temperature of 22 °C and 50% humidity for 28 days. After the seasoning period, the glued composites were cut to align the edges of the samples after gluing. At the next stage, the surfaces of the samples were examined. The geometries of the composites were tested with a mechanical plotter from the facing side of the composites. The accuracy of the measurement was 0.1 mm. The differences in the flatness of the samples did not exceed +/− 3 mm. Examples of the prepared samples are presented in Figure 2.

The results of the experimental samples were compared with those of the industrial samples. The base layers of the industrial composites were achieved via sawing using a multi-blade sawing machine. The thicknesses of the tested and industrial base layers were the same.

### 2.2. Tests of Static Bending

**Figure 3:**
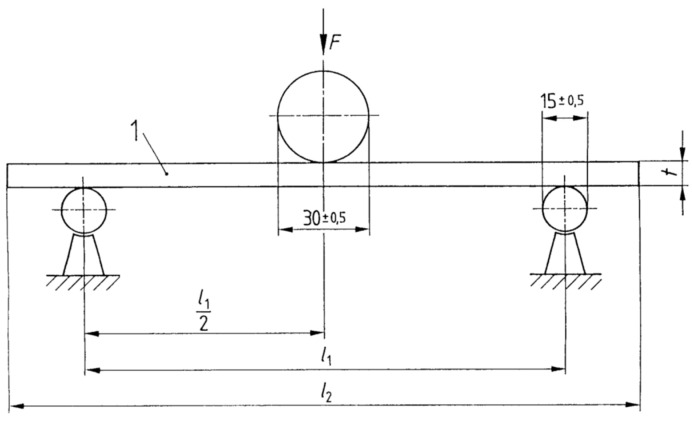
Arrangement of the bending apparatus: l_1_—310 mm, l_2_—340 mm, 1—sample and t—sample thickness.

The test of the modulus of elasticity was carried out on a TiraTest 2300 (TIRA GmbH, Schalkau, Germany) universal testing machine using a three-point scheme based on the standard EN 310: 1993 [33], as presented in Figure 3. In this article, Young’s modulus—the modulus of elasticity (MOE) and stiffness—was considered the most useful value.

The samples were placed on supports with a spacing of 310 mm and were subjected to a force of 2 kN. The modulus of elasticity in static bending was calculated on the basis of the formula:

(1) $$E_{m}=\frac{l_{1}^{3}(F_{2}-F_{1})}{4bt^{3}(a_{2}-a_{1})}$$

where

E_m_ [MPa]—modulus of elasticity;

l [mm]—distance between support centres;

F_2_ [N]—40% of maximum force;

F_1_ [N]—10% of maximum force;

b [mm]—sample width;

t [mm]—sample thickness;

a_2_ − a_1_ [mm]—deflection arrow increment measured at the middle of the sample length (corresponding to F_2_ − F_1_).

Stiffness is the ability of an element, joint or structure to resist deformation due to an external load. A material’s stiffness depends on its shape, elastic properties, type of loads and boundary conditions. One of the methods of determining dynamic stiffness is discussed in the ISO 9052-1 standard [34]. However, this standard presents a different methodology and loads. Therefore, in order to be able to compare the results, the stiffness of composites under static and dynamic loading conditions was determined on the basis of the formula:

(2) $$k=\frac{E_{m}*b*t^{3}}{12}$$

where

k [MNmm^2^]—stiffness;

E_m_ [MPa]—modulus of elasticity;

b [mm]—sample width;

t [mm]—sample thickness.

### 2.3. Shewhart’s Theory Focused on the Subject of the Work

One of the methods used in industry for statistical inspections of the technical parameters of a product is the Shewhart control chart without set normative values [35]. Depending on the type of numerical data collected, the Shewhart control chart can be used to characterise measured features of the process/analysis. The most frequently chosen measure in analyses is the average of the tests performed, which determines the value of the technical parameter to which individual measurements are compared. The research analysis is carried out on standardised cards, though various authors [36,37] have proposed using sequential cards that differ regarding several aspects.

An important element of standard Shewhart control charts are the lines drawn on the chart. The central line (target) corresponds to the expected value of the process being inspected. Its estimator is the sample mean. Lower and upper control lines (LCL and UCL) are drawn parallel to the centre line, which is preceded by lower and upper specification lines (LSL and USL) at a distance [38]. Specification lines are determined according to the formula:

(3) SL=Target±2δ

where

SL—specification line value;

Target—mean value for group of tested samples;

δ—standard deviation determined for industrial samples.

Control Lines were determined according to the formula:

(4) CL=Target±3δ

where

CL—control line value;

Target—mean value for group of tested samples;

δ—standard deviation determined for industrial samples.

The control lines define the allowable deviation from the centre line of the individually measured parameters. Exceeding them in production technology signifies the production of a defective product. Specification lines are necessary to control the stability of the technological process. A large number of measurements located between the control and specification lines indicate that the process parameters need to be corrected.

In order for the tested product parameter to be analysed using Shewhart control charts, the measurements must meet certain conditions. Assuming that the distribution of the parameter values characterising a feature of the controlled process is Gaussian (normally distributed), the control lines limit the area on the card in which 99.7% of the value of this parameter lies. In other words, the probability of a parameter value being between the control lines is 0.997 [39]. If the presented condition is met by the measurements performed, it is possible to analyse samples of individual quality classes using the Shewhart control chart.

The industrial samples were analysed first in order to obtain the minimum values of Young’s modulus and stiffness that should be achieved by experimental samples of all classes. Based on the data obtained, fixed ranges for control lines and specification lines were also determined. For each new card, a comparison was made between the average value of the series of results placed on the card and the expected value for a given class. If there was a significant difference between the compared values, the received card was discarded.

In the last step, a Shewhart control chart was created for all experimental samples tested. In this case, the normative values were determined on the basis of the measurement results obtained in the study (after 30 repetitions). On this basis, the value of the overall mean (target) of the single analysis and the mean value of the standard deviation were determined.

## 3. Results and Discussion

**Figure 4:**
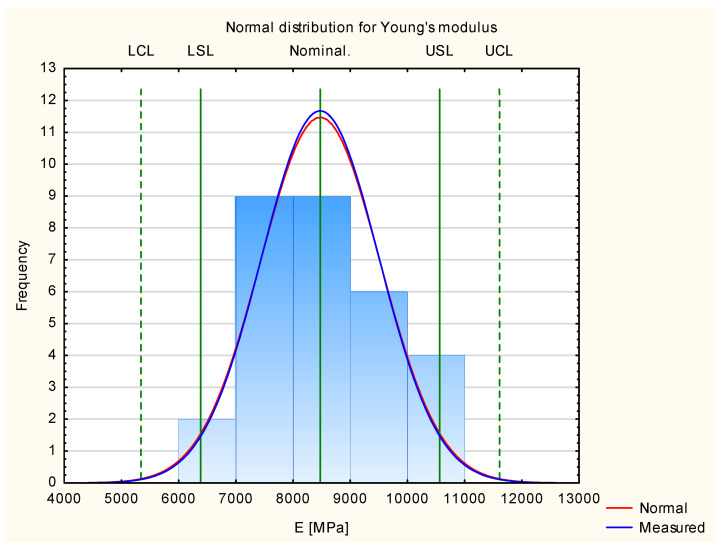
Normal distribution of Young’s modulus.

Table 1 shows all the measurement results from the experimental samples. The last line presents the results of the calculated statistical data used to create a normal distribution chart (presented in Figure 4), which determines whether it is possible to conduct an analysis using the Shewhart control chart method. All the Young’s modulus values at this stage were calculated for all test results of all the experimental samples. According to the described methodology, an analysis of the compliance of the normal distribution of all the experimental results and the conditions of the six sigma concept is an essential element used to determine the correctness of the adopted quality control implementation methodology [40].

### 3.1. Young’s Modulus

Figure 4 presents the results for Young’s modulus for all the tested experimental samples. It shows that the results are appropriate for normal distribution. More than 99.7% of the results are within the frame of six sigma (all of the measured samples are situated between the border lines LCL and UCL). This allows us to proceed with Shewhart’s theory.

Table 2 presents the results of the calculated indices used to create Shewhart control charts of Young’s modulus for each sample group. In order to compare the stability of the sample properties, a constant standard deviation value was established on the basis of industrial samples. Industrial samples were characterised with the lowest dispersion of the results, which ensures certainty and comparability of process stability for samples made of different wood quality classes. On this basis, control lines and specification lines were established.

**Figure 5:**
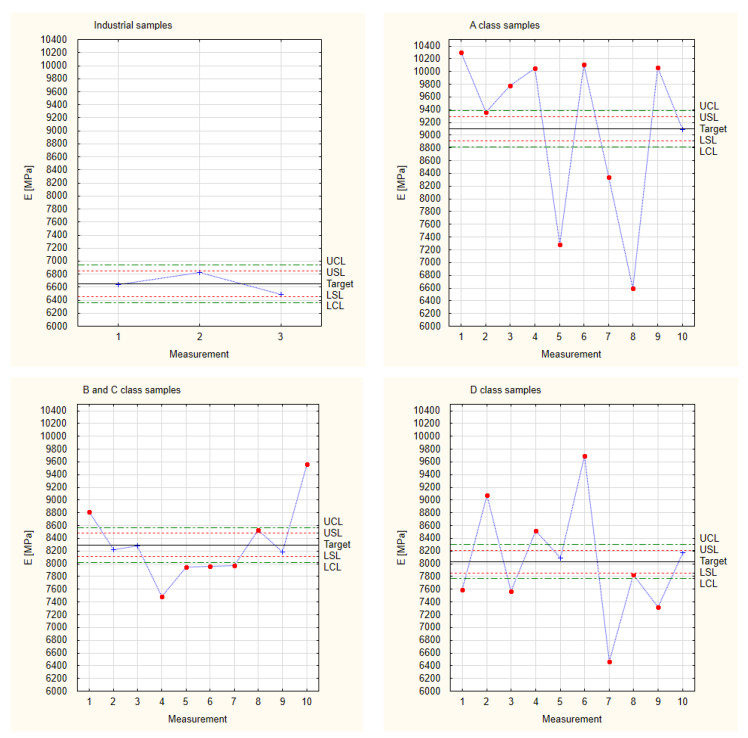
Shewhart control charts of Young’s modulus.

Figure 5 shows the results calculated for Young’s modulus based on Shewhart’s theory. Three industrial samples were taken for analysis because industrial production has been continuously developed over the years, so there is no need to analyse a large number of samples. At first glance, it is clear that the tested industrial samples have very comparable results, thus proving the accuracy and precision of the workmanship. All the obtained results are within the adopted specification lines (LSL and USL). This is a clear result of the way these industrial samples are produced. At the production stage, defective samples are rejected, and further processing ceases. In addition, the chip sawing production method using multi-saws enables in situ process control. The experimental samples made by hand show a large scatter for the results of Young’s modulus, regardless of the quality class of the material used. These samples were obtained from experimental production, i.e., without the possibility of exact repeatability. Nevertheless, the target values, regardless of the quality class of the wood used, are higher than the target values for industrial samples. This means that the material can be used to produce composites without rejecting parts of it due to low quality class. 

**Figure 6:**
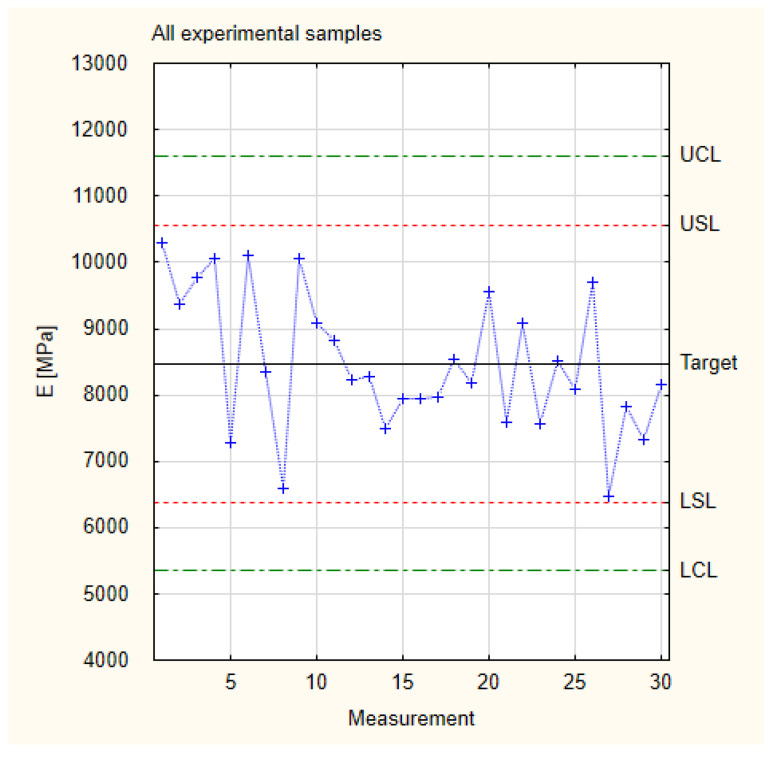
Collected test results of experimental samples for Young’s modulus.

Since all the experimental sample targets have values higher than the industrial samples, they can be grouped into one plot. These values are presented in Figure 6, which is based on the measurement values and calculations of Young’s modulus presented in Table 1. The total target of experimental samples is within the range of 8500 MPa. This value is approximately 30% higher than the industrial target. It is also worth noting that the lowest Young’s modulus values for the experimental samples were higher than the target for the industrial samples and that all of the obtained measurement values fell between the designated specification lines, thus achieving the aim of the experiment.

### 3.2. Stiffness

**Figure 7:**
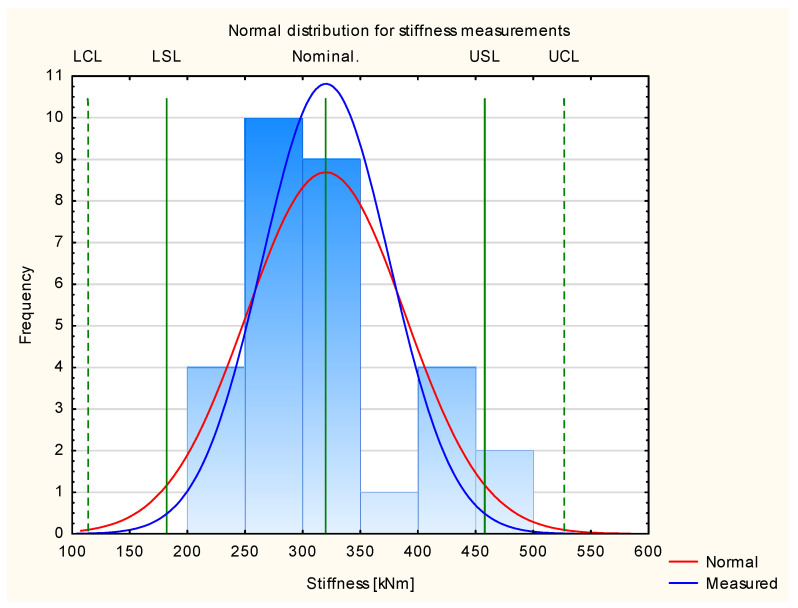
Normal distribution of stiffness.

Figure 7 presents the results for stiffness for all the tested experimental samples. It shows that the results are normally distributed. More than 99.7% of the results are in the frame of six sigma (all of the measured samples are situated between the border lines LCL and UCL). This allows us to proceed with Shewhart’s theory.

Table 3 presents the results of the calculated indices used to create Shewhart control charts of stiffness for each sample group. In order to compare the stability of the sample properties, a constant standard deviation value was established and calculated for the industrial samples. On this basis, control lines and specification lines were established.

**Figure 8:**
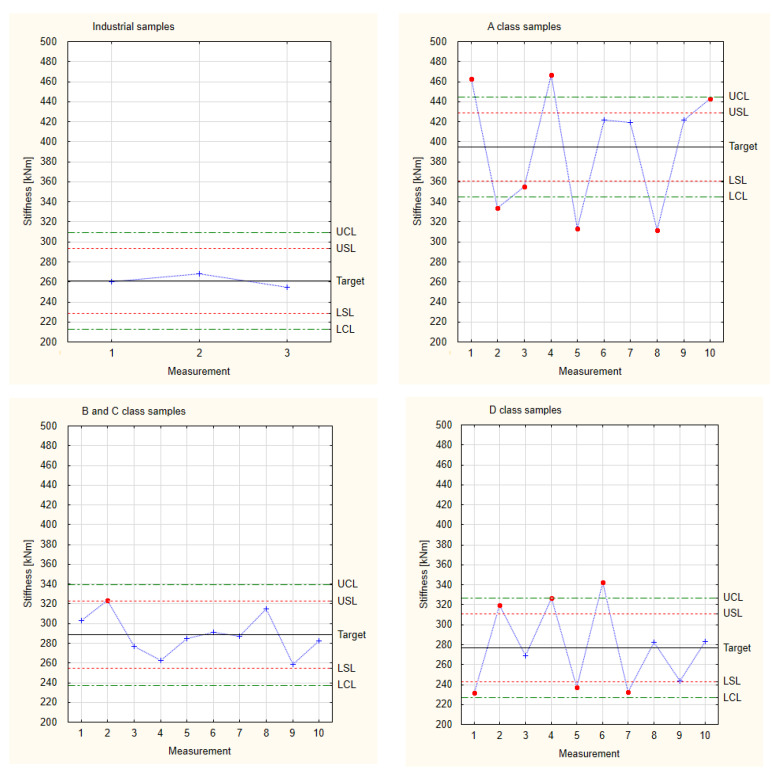
Shewhart control charts of stiffness.

Figure 8 shows the results calculated for stiffness in relation to Shewhart’s theory. As in the case of the results obtained for Young’s modulus, the industrial samples are within the specification lines, while the experimental samples show a wide range of results. This is especially visible for the highest and lowest classes of wood used. However, the samples made from the mixed B and C class wood have values with the highest repeatability and are within the assumed industrial quality class. This resulted from a qualitative visual assessment. In this evaluation, A class samples can be mixed with B class samples, and D class samples can be mixed with C class samples. Nevertheless, the experimental samples show target values that are higher than the target values of the industrial samples.

**Figure 9:**
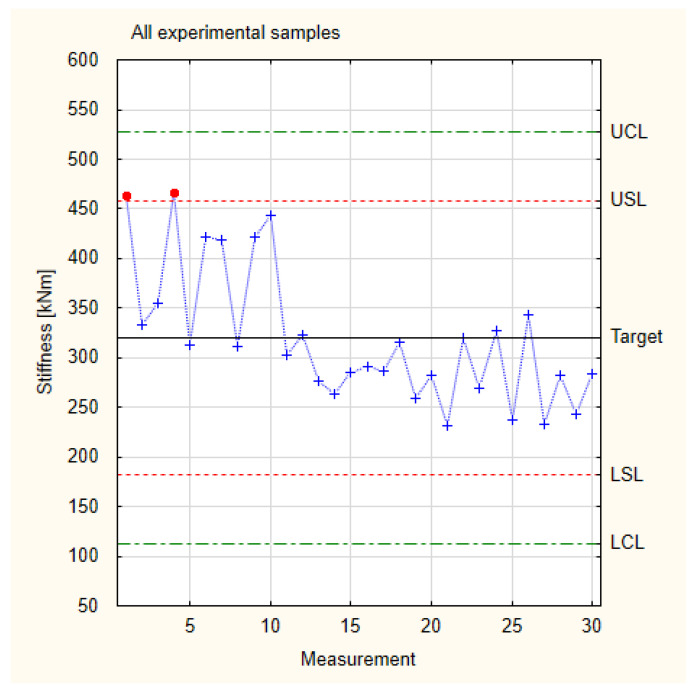
Collected test results of the experimental samples for stiffness.

Since all the experimental samples have higher target stiffness values than the industrial samples, they can be analysed together. The collected stiffness values of the experimental samples are presented in Figure 9, which is based on the measurement values and calculations for stiffness presented in Table 1. Although the experimental samples were produced manually, the values obtained for them are close to the LSL. This means that an improvement in technology from manual to mechanical would definitely change the spread of the results so that the values would be within the control lines and also very likely within the specification lines.

## 4. Conclusions

A base layer in composites for flooring materials made of veneers in plywood, like the structure achieved through rotary cutting, had better mechanical properties than a base layer prepared using the sawing process, which was expected. Surprisingly, the mechanical properties of veneer composites made only of the lowest quality veneers (class D) have higher values—about 20% for Young’s modulus and 8% for stiffness. Even higher strength values have been achieved using base layers of veneers from mixed classes (A/B/C/D) compared to industrial products (30% and 23%, respectively).

Production without quality selection has only benefits: no material waste and no time-consuming or cost-intensive quality control. Research has shown that production is predicted in relation to Shewhart’s theory. At the same time, it should be noted that, despite the many advantages of using Shewhart control charts in analyses of the parameters of a manufactured product, these charts are rarely used in scientific research analysis in the field of woodworking [41]. Most quality analyses of wooden elements are based on individual standards for a specific parameter being tested [42,43].

High-value mechanical parameters enable the cross-arrangement of veneers in the base layer, which is impossible when production is cutting-based. This is the experimental procedure for handmade composites, which results in unique technological parameters. Additionally, the production of composites for flooring materials based on rotary cutting is chipless. Residues of this type of production, e.g., in the form of a regular cylinder, are very easy to use in manufacturing other goods.

## Figures and Tables

**Table 1:** Experimental test results.

Sample Group	Young’s Modulus [MPa]	Stiffness [kNm]
A class	10,300	463
	9364	334
	9778	355
	10,048	467
	7278	313
	10,115	422
	8337	419
	6597	312
	10,058	422
	9087	443
B/C class	8816	303
	8224	323
	8275	277
	7482	263
	7950	285
	7955	291
	7977	287
	8532	315
	8185	259
	9559	282
D class	7597	232
	9081	320
	7567	269
	8519	327
	8089	237
	9697	343
	6468	232
	7834	282
	7324	243
	8169	283
Standard deviation	1043	69
Lower Control Line	5345	113
Lower Specification Line	6388	182
Nominal (average value)	8475	320
Upper Specification Line	10,562	458
Upper Control Line	11,605	527

**Table 2:** Young’s modulus summary of values for Shewhart control chart [MPa].

Sample Group	Standard Deviation for All Samples	LCL	LSL	Nominal	USL	UCL
Industrial	98	6357	6455	6651	6847	6945
A class		8802	8900	9096	9292	9390
B/C class		8002	8100	8296	8492	8590
D class		7741	7839	8035	8231	8329

**Table 3:** Stiffness summary of values for Shewhart control chart [kNm].

Sample Group	Standard Deviation	LCL	LSL	Nominal	USL	UCL
Industrial	17	209	226	260	294	311
A class		344	361	395	429	445
B and C class		238	255	289	323	340
D class		227	244	278	311	328

## Data Availability

Data are contained within the article.
